# CHIP protects against MPP^+^/MPTP-induced damage by regulating Drp1 in two models of Parkinson’s disease

**DOI:** 10.18632/aging.202389

**Published:** 2021-01-02

**Authors:** Zhengwei Hu, Chengyuan Mao, Hui Wang, Zhongxian Zhang, Shuo Zhang, Haiyang Luo, Mibo Tang, Jing Yang, Yanpeng Yuan, Yanlin Wang, Yutao Liu, Liyuan Fan, Qimeng Zhang, Dabao Yao, Fen Liu, Jonathan C. Schisler, Changhe Shi, Yuming Xu

**Affiliations:** 1 Department of Neurology, The First Affiliated Hospital of Zhengzhou University, Zhengzhou University, Zhengzhou, Henan, China; 2 The Academy of Medical Sciences of Zhengzhou University, Zhengzhou University, Zhengzhou, Henan, China; 3 Sino-British Research Centre for Molecular Oncology, National Centre for International Research in Cell and Gene Therapy, School of Basic Medical Sciences, Academy of Medical Sciences, Zhengzhou University, Zhengzhou, Henan, China; 4 McAllister Heart Institute at The University of North Carolina at Chapel Hill, Chapel Hill, NC 27599, USA; 5 Department of Pharmacology, and Department of Pathology and Lab Medicine at The University of North Carolina at Chapel Hill, Chapel Hill, NC 27599, USA; 6 Henan Key Laboratory of Cerebrovascular Diseases, The First Affiliated Hospital of Zhengzhou University, Zhengzhou, Henan, China; 7 Institute of Neuroscience, Zhengzhou University, Zhengzhou, Henan, China

**Keywords:** Parkinson’s disease, CHIP, Drp1, MPTP, gene knockin

## Abstract

Mitochondrial dysfunction has been implicated in the pathogenesis of Parkinson’s disease (PD). Carboxyl terminus of Hsp70-interacting protein (CHIP) is a key regulator of mitochondrial dynamics, and mutations in CHIP or deficits in its expression have been associated with various neurological diseases. This study explores the protective role of CHIP in cells and murine PD models. In SH-SY5Y cell line, overexpression of CHIP improved the cell viability and increased the ATP levels upon treatment with 1-methyl-4-phenylpyridinium (MPP^+^). To achieve CHIP overexpression in animal models, we intravenously injected mice with AAV/BBB, a new serotype of adeno-associated virus that features an enhanced capacity to cross the blood-brain barrier. We also generated gene knock-in mice that overexpressed CHIP in neural tissue. Our results demonstrated that CHIP overexpression in mice suppressed 1-Methyl-4-phenyl-1,2,3,6-tetrahydropyridine (MPTP)-induced damage, including movement impairments, motor coordination, and spontaneous locomotor activity, as well as loss of dopaminergic neurons. *In vitro* and *in vivo* experiments showed that overexpression of CHIP inhibited the pathological increase in Drp1 observed in the PD models, suggesting that CHIP regulates Drp1 degradation to attenuate MPP^+^/MPTP-induced injury. We conclude that CHIP plays a protective role in MPP^+^/MPTP-induced PD models. Our experiments further revealed that CHIP maintains the integrity of mitochondria.

## INTRODUCTION

Parkinson’s disease (PD), which is characterized by clinical features such as bradykinesia, tremor, rigidity, and autonomic dysfunction, is the second most common neurodegenerative disease [1]. Progressive aggregation of Lewy bodies in the cytoplasm and loss of dopaminergic neurons in the substantia nigra pars compacta (SNpc) are the main pathological manifestations of PD. While gene mutations and environmental influences across aging are thought to contribute to the development of PD, the exact mechanisms of PD onset remain unclear [2].

Mitochondrial dysfunction is considered the core factor in the pathogenesis of PD. Mitochondria are highly dynamic organelles essential for energy conversion, and the high energy demand of neurons renders them vulnerable to mitochondrial dysfunction [3]. Indeed, disruption of mitochondrial dynamics has been implicated in the pathogenesis of several neurodegenerative diseases, including Alzheimer’s disease, Charcot-Marie-Tooth disease, and Huntington's disease [4–6]. Mitochondrial homeostasis is in part maintained by the balance between mitochondrial fission and fusion, and damage to mitochondrial homeostasis leads to excessive production of reactive oxygen species and decreased membrane potential [7]. Accumulating evidence from *in vivo* and *in vitro* PD models shows that modulation of mitochondrial dynamics can attenuate PD-associated impairments. Thus, a potential avenue of PD treatment involves preserving the normal function of mitochondria [8].

The carboxyl terminus of Hsp70-interacting protein (CHIP) is a co-chaperone protein essential for protein quality control. In addition, CHIP is considered to be a central molecular player in mitochondrial stress signaling and cellular homeostasis. CHIP dysfunction has been implicated in various neurological diseases [9]. CHIP also interacts with α-synuclein, Parkin, and LRRK2, further suggesting that CHIP participates in PD-associated pathways [10–12]. CHIP knockout animals exhibit movement disorders, and electron-microscopic examination has revealed that these models have higher numbers of mitochondria with swollen and misshapen morphology than do the wild-type controls. Mutations in PTEN-induced putative kinase 1 (PINK1) contribute to the pathogenesis of PD. CHIP promotes PINK1 ubiquitination and thus negatively regulates PINK1 stability [13]. Overexpression of CHIP in *Pink1*-mutated *Drosophila* ameliorated locomotion impairments, loss of dopaminergic neurons, and mitochondrial defects [14]. Furthermore, CHIP is considered protective against neurotoxic stimuli, and the regulation or modification of CHIP can affect the shape of mitochondria, thereby changing the cellular response to stress [15].

1-Methyl-4-phenyl-1,2,3,6-tetrahydropyridine (MPTP) has been widely used to generate models of PD. After crossing the blood-brain barrier, MPTP is metabolized to 1-methyl-4-phenylpyridinium (MPP^+^), which damages the mitochondrial respiratory chain in dopaminergic neurons and eventually leads to cell death [16]. MPP^+^-treated human neuroblastoma cells have been used as cellular models of PD *in vitro*. To explore the protective role of CHIP in PD models, we used adeno-associated virus (AAV) to upregulate the expression of CHIP in adult mice. MPTP was then injected to induce PD-like symptoms. AAV/BBB is a new variant of AAV that can cross the blood-brain barrier with high efficiency, enabling effective intravenous gene transfer to the mouse central nervous system. We also generated a gene knock-in mouse model based on Nestin-cre/loxP-driven CHIP overexpression that allows for the selective induction of CHIP in neural tissue. We then studied the effect of CHIP overexpression *in vivo* on the amelioration of motor impairments, cell viability, and mitochondrial homeostasis in MPTP-induced PD models. Our results may provide new evidence for the protective effect of CHIP in PD and other neurodegenerative diseases.

## RESULTS

### CHIP attenuated cell damage caused by MPP^+^ and modulated the Bcl-2/Bax ratio

**Figure 1:**
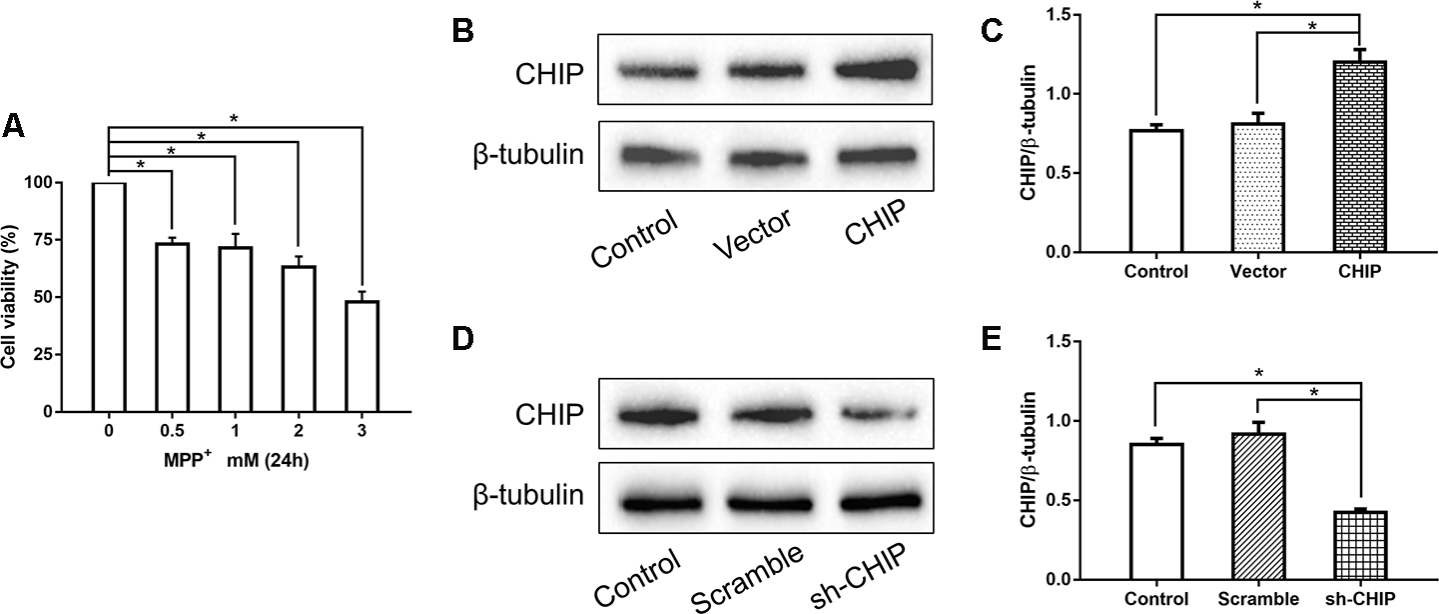
**Concentration screening of MPP^+^ and verification of CHIP plasmid and shRNA.** (**A**) SH-SY5Y cells were treated with different concentrations of MPP^+^ to determine the optimum concentration. (**B**, **C**) Western blotting showed CHIP increased significantly after transfection with CHIP plasmid. (**D**, **E**) The expression of CHIP decreased under the action of shRNA. *P<0.05.

**Figure 2:**
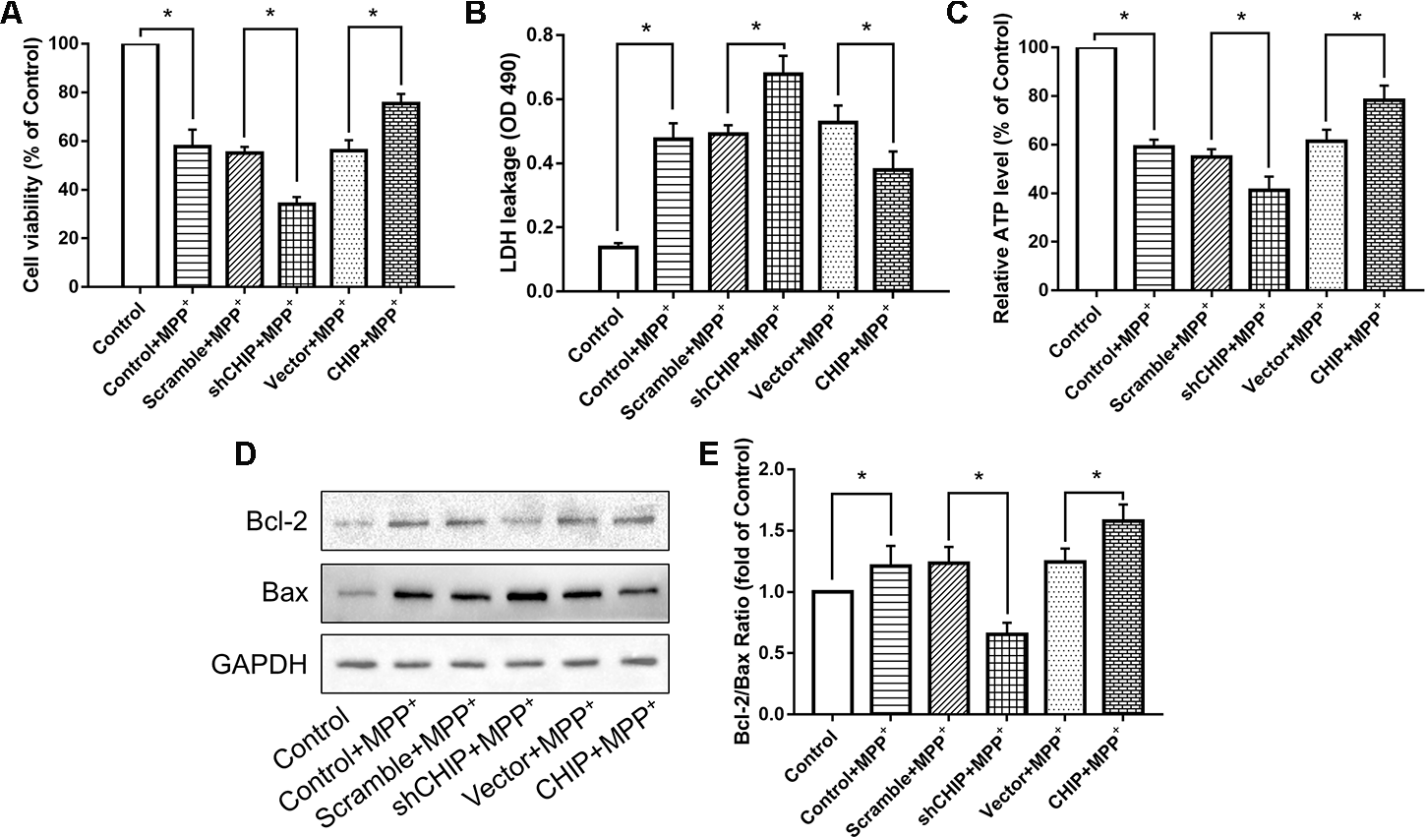
**CHIP improved cell damage caused by MPP^+^ and modulated Bcl-2/Bax Ratio.** (**A**–**C**) Overexpression of CHIP before MPP^+^ induced increased cell viability, decreased LDH level and increased ATP level. Decreased CHIP played the opposite role. (**D**, **E**) The increased Bcl-2/Bax ratio caused by MPP^+^ was inhibited in CHIP-overexpression group, while in sh-CHIP group, the ratio was even higher. *P<0.05.

Human neuroblastoma (SH-SY5Y) cells were treated with different concentrations of MPP^+^ to determine the optimum concentration for generating the PD model *in vitro*. According to the results of a cell viability assay, 2 mM of MPP^+^ was sufficient to induce cell damage (Figure 1A). Western blotting proved the CHIP plasmid and the shRNA to be effective (Figure 1B–1E). To detect the effect of CHIP on MPP^+^-induced cell damage, we assessed cell viability by kit and cell damage as lactate dehydrogenase (LDH) leakage. After treatment with 2 mM of MPP^+^ for 24 h, we observed an obvious cell viability decrease and extracellular LDH increase. Overexpression of wild-type CHIP prior to MPP^+^ exposure increased cell viability and decreased LDH levels relative to the vector control (Figure 2A, 2B). We also examined these two indicators in CHIP-deficient (transfected with CHIP-shRNA) cells after MPP^+^ exposure and found the cells to be significantly more sensitive to MPP^+^-induced injury (Figure 2A, 2B).

Levels of ATP were also measured. We found that overexpression of CHIP prior to MPP^+^ exposure resulted in enhanced ATP levels, while reduced ATP production was observed in cells transfected with CHIP-shRNA, compared to the ATP levels in controls with endogenous CHIP levels (Figure 2C). This evidence indicated that CHIP plays a neuroprotective role during MPP^+^-induced injury.

We found that MPP^+^ increased the level of apoptosis in wild-type SH-SY5Y cells. A key indicator of mitochondria-induced apoptosis is lowered Bcl-2/Bax ratio. Overexpression of CHIP significantly increased the Bcl-2/Bax ratio post MPP^+^ exposure, while CHIP knockdown decreased this ratio (Figure 2D, 2E). These results suggest that enhance CHIP activity can reduce apoptosis in MPP^+^-damaged SH-SY5Y cells.

### CHIP prevented the MPP^+^-induced upregulation of Drp1

**Figure 3:**
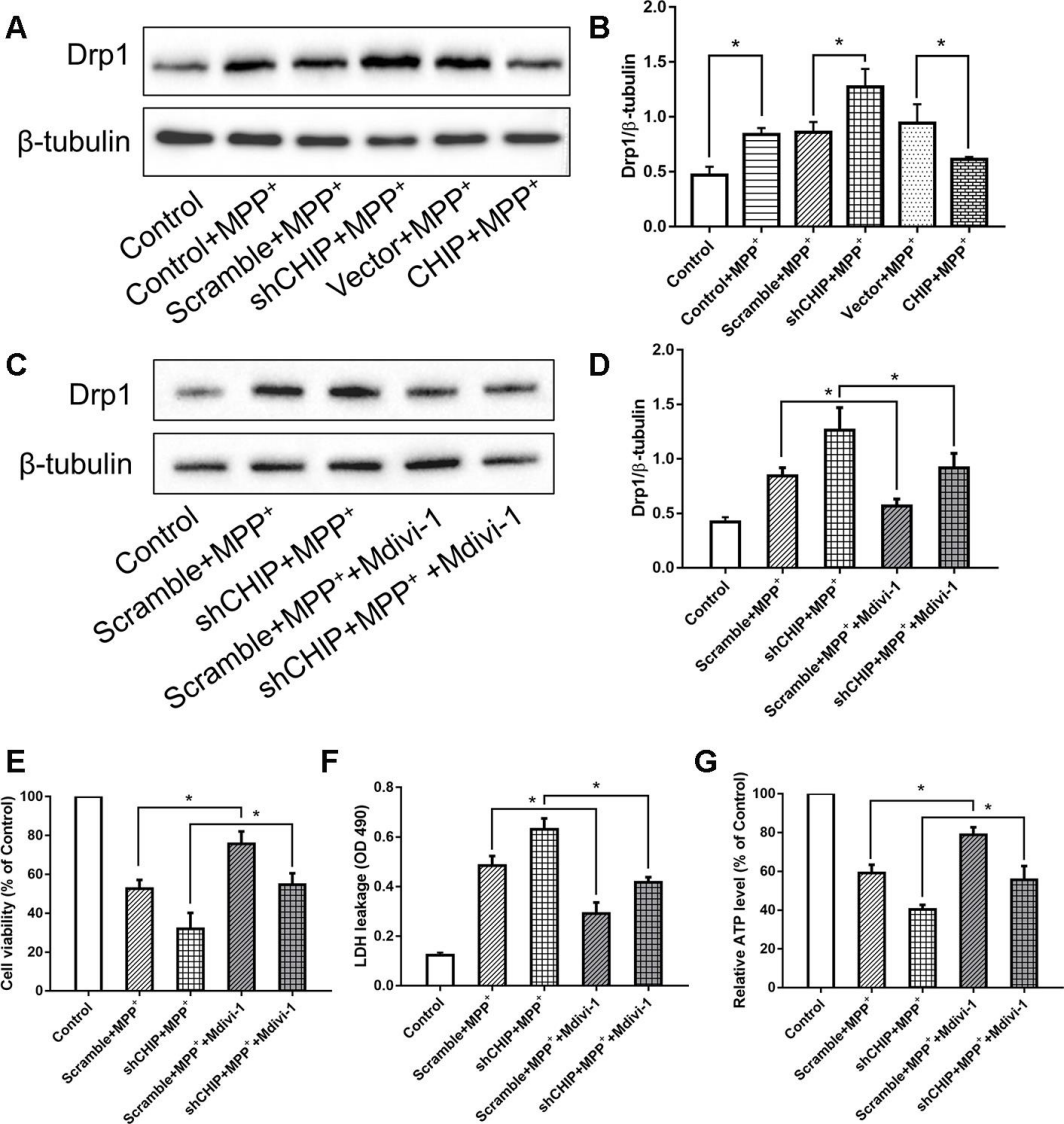
**CHIP prevented the upregulation of Drp1 induced by MPP^+^.** (**A**, **B**) Immunoblot of Drp1 in different groups of MPP^+^-induced PD cell models. MPP^+^ caused the increase of Drp1 in SH-SY5Y cells. After transfecting with CHIP plasmid, the rising trend of Drp1 was inhibited. (**C**, **D**) Drp1 levels in MPP^+^-induced cells with or without Mdivi-1 treated. Mdivi-1 could prevent the increase of Drp1 caused by MPP^+^. (**E**–**G**) Under the condition of CHIP knockdown, LDH and ATP levels in MPP^+^-induced cells with or without Mdivi-1 treated were detected. *P<0.05.

Consistent with other research, our results showed that MPP^+^ induced an increase in Drp1. We investigated the regulatory effect of CHIP on Drp1 levels in SH-SY5Y cells post-MPP^+^ treatment. We found that CHIP overexpression prevented the high Drp1 levels induced by MPP^+^, and CHIP knockdown further increased Drp1 levels after MPP^+^ injury (Figure 3A, 3B). After transfection with CHIP-shRNA, we treated cells with MPP^+^ followed by Mdivi-1, an inhibitor of Drp1. The increase in Drp1 and LDH leakage normally observed post MPP^+^ were abolished in cells exposed to Mdivi-1. Further, cell viability and ATP production were obviously increased (Figure 3C–3G). Taken together with the data described in previous subsections, these findings indicate that CHIP prevents the increase in Drp1 induced by MPP^+^.

### CHIP ameliorated the mitochondrial fragmentation caused by MPP^+^

**Figure 4:**
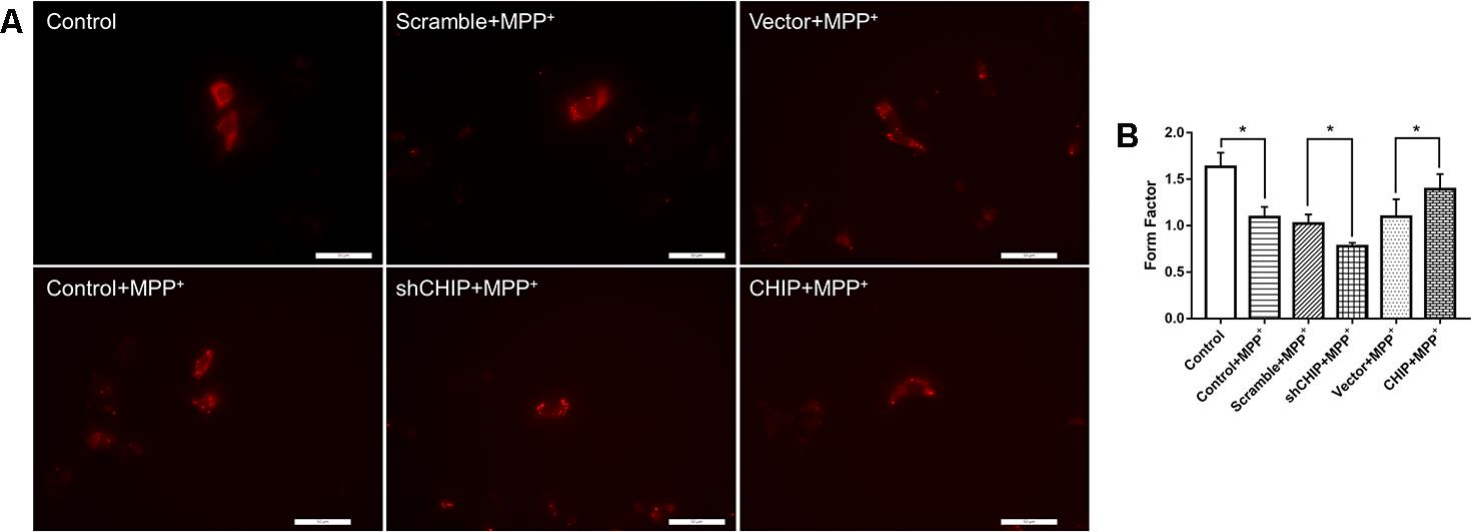
**Mitochondrial morphology change of each group.** (**A**) Mitochondria were labeled with MitoTracker Red. MPP^+^ treatment lead to fragmentation of mitochondria, while CHIP overexpression improved the mitochondria disruption. (**B**) Histogram of form factor analysis. *P<0.05.

After treatment with 2 nM MPP+ for 24 h, SH-SY5Y cells showed broken mitochondrial reticulata and increased debris after MitoTracker Red (MTR) staining and analysis of the mitochondrial form factors. Greater numbers of mitochondrial fragments were detected in CHIP-knockdown cells, while in CHIP-overexpressing cells, the extent of mitochondrial fragmentation was less severe than in cells receiving only MPP^+^ (Figure 4A, 4B).

### Overexpression of CHIP in two mouse models: AAV transfection and CHIP knock-in

**Figure 5:**
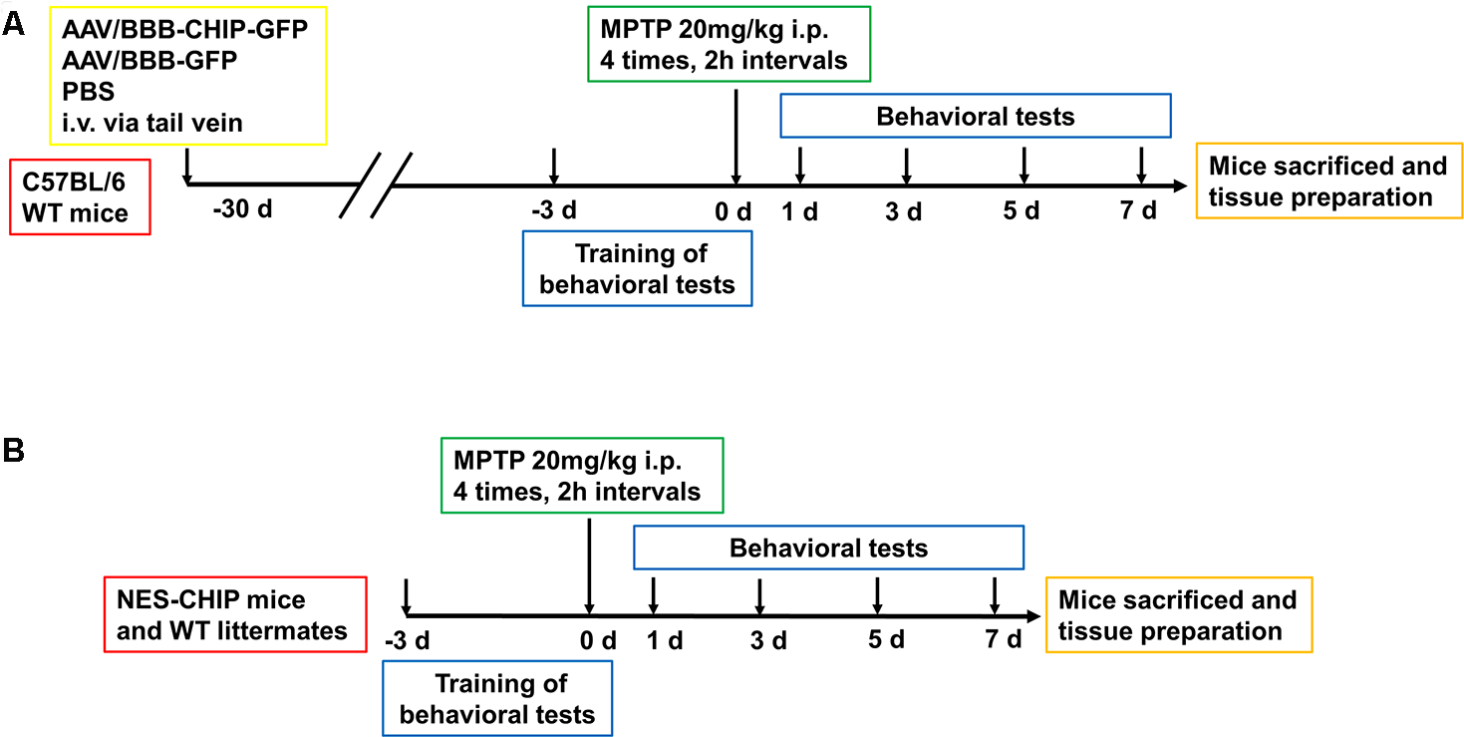
**Experimental design of *in vivo* experiment.** (**A**) C57BL/6 wildtype mice were divided into three groups, 8-10 mice per group. At 6–8 weeks old, mice received equal dose of AAV or PBS injection intravenously via tail vein. 4 weeks later, mice were trained for behavioral tests including pole test, rotarod test and open-field test. Then animals were administered four intraperitoneal injections of MPTP (20 mg/kg) at 2 h intervals. Behavioral tests were conducted at 1^st^, 3^rd^, 5^th^ and 7^th^ days. After that, Mice were sacrificed for the continue experiment. (**B**) NES-CHIP mice and wildtype littermates were treated in the same way with the former animals except for not receiving injections.

**Figure 6:**
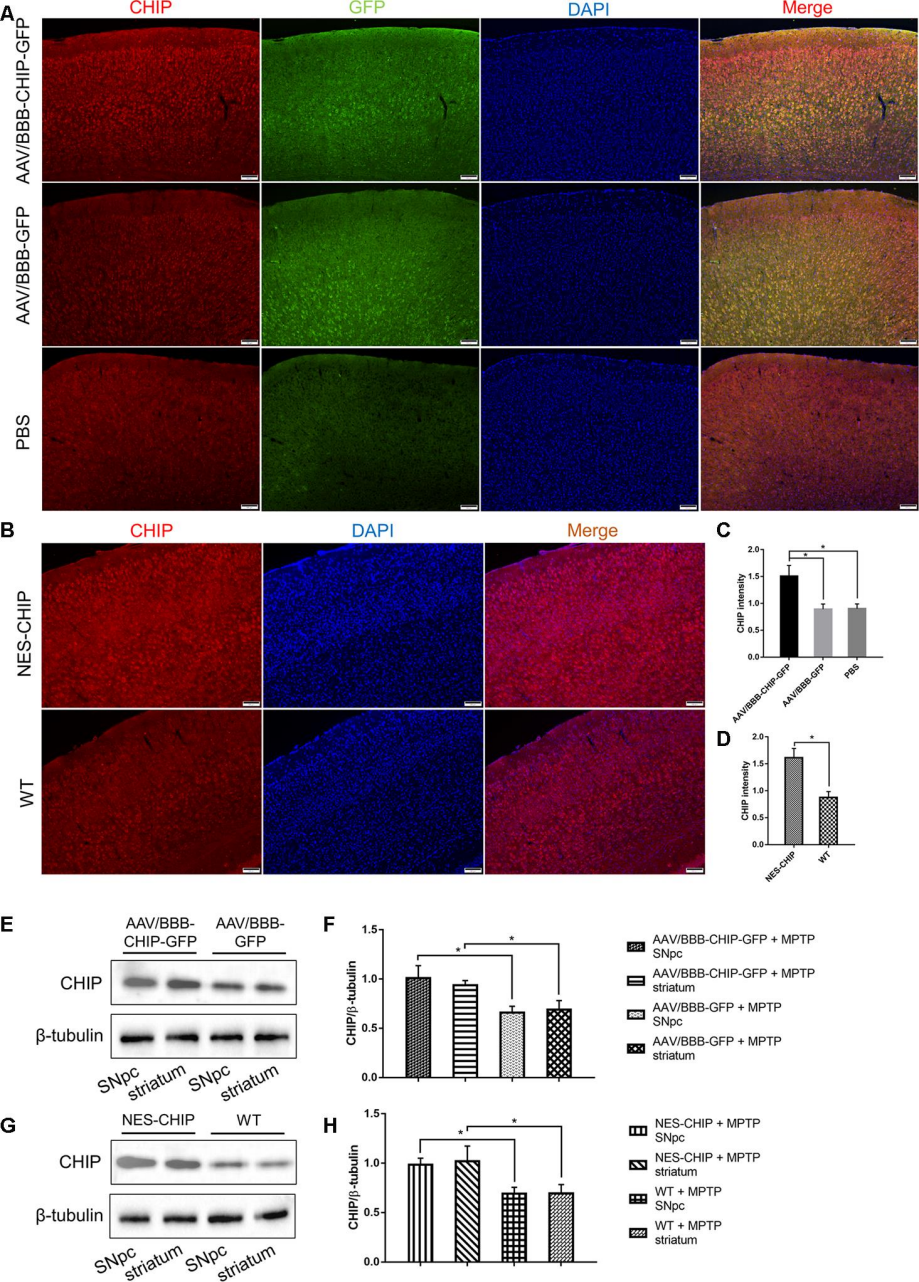
**Overexpression of CHIP in AAV-mediated transduction mice and NES-CHIP mice.** (**A**, **C**) Immunofluorescence for CHIP (red), GFP (green) and nucleus (blue) showed that CHIP overexpressed in mice injected with AAV/BBB-CHIP-GFP. (**B**, **D**) In NES-CHIP mice, immunofluorescence proved a higher level of CHIP (red) than that in wild-type littermates. (**E**–**H**) Western blotting showed increased CHIP level in SNpc and striatum of mice received AAV/BBB-CHIP-GFP injection and NES-CHIP. CHIP *P<0.05.

The design of the animal experiments is shown in Figure 5. To overexpress CHIP, we injected AAV/BBB-CHIP-GFP, the vector control AAV/BBB-GFP, or the same volume of vehicle into wild-type mice via the tail vein at 6–8 weeks of age. Four weeks later, the mice were sacrificed, and the expression of CHIP was verified in brain slices and protein extracts. We also used gene knock-in mice carrying an exogenous *STUB1* gene to achieve neural CHIP expression. NES-CHIP mice and wild-type littermates aged 10–12 weeks were chosen for the experiment. The distribution and expression of proteins were assessed with CHIP/GFP double staining in the AAV group and with CHIP immunofluorescence in the knock-in group. Compared with the mice that received vehicle, mice that received AAV/BBB-CHIP-GFP or AAV/BBB-GFP showed increased expression of GFP. However, CHIP was significantly upregulated only in the AAV/BBB-CHIP-GFP group. Similar results were confirmed in the NES-CHIP mice (Figure 6A, 6C). The level of CHIP detected was lower in wild-type animals than in their knock-in littermates (Figure 6B, 6D). Western blotting confirmed these trends in CHIP expression (Figure 6E–6H).

### CHIP overexpression ameliorated MPTP-induced motor impairments

**Figure 7:**
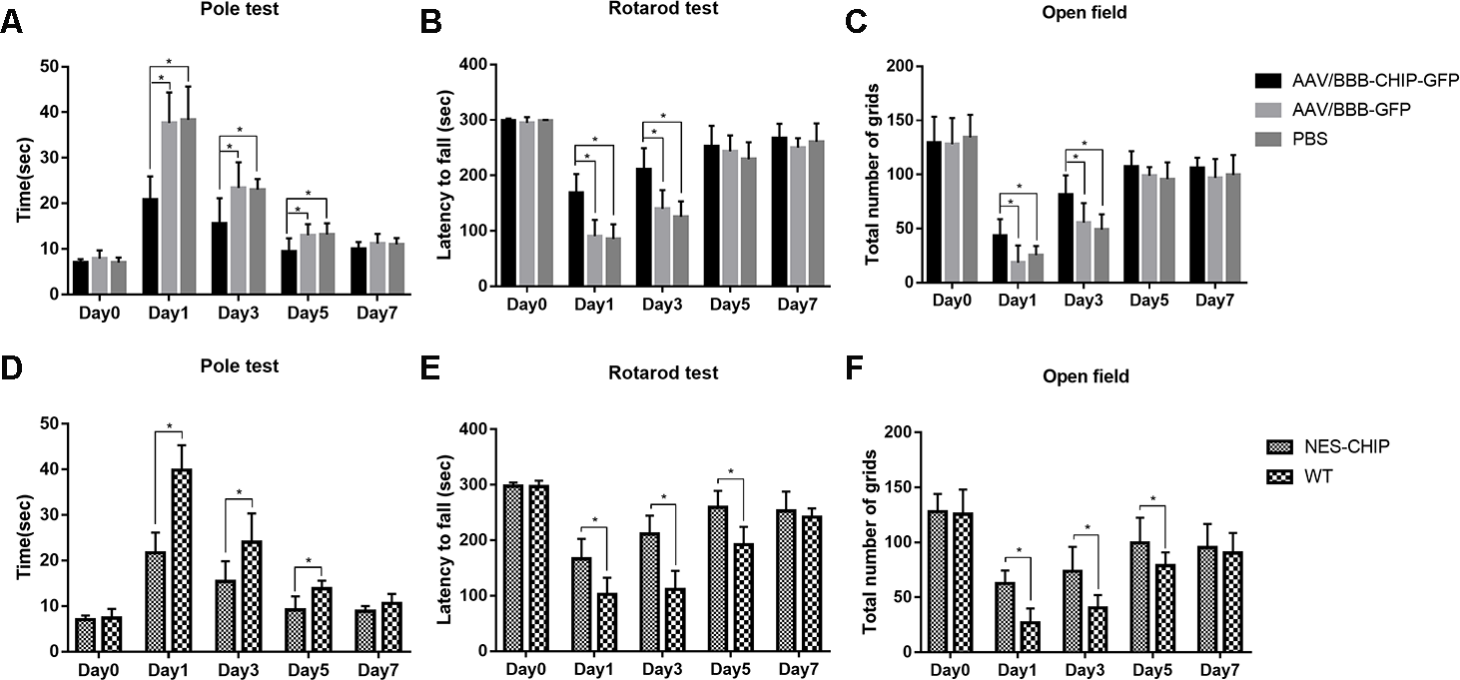
**CHIP overexpression ameliorated MPTP-induced motor impairments in mice.** Pole test, rotarod test and open field test were performed to evaluate motor function of each group. (**A**, **D**) Total time the mice used to climb from top of the pole. (**B**, **E**) Latency to fall in rotarod test after MPTP administration. (**C**, **F**) Number of grids the mice crossed in 5 minutes in the open field test. *P<0.05.

We established MPTP-induced PD models to determine whether CHIP overexpression could ameliorate PD-like symptoms. Behavioral tests were performed at baseline and on the 1^st^, 3^rd^, 5^th^, and 7^th^ days after the last injection of MPTP. Movement disorders, motor coordination, and spontaneous locomotor activity were assessed with the pole test, accelerating rotarod, and open-field test, respectively. The baseline data showed no significant differences among groups. MPTP exposure diminished motor ability in all groups. Overexpression of CHIP prevented drug injury and ameliorated the motor impairments. Compared with controls and wild-type animals, mice pre-treated with AAV/BBB-CHIP-GFP and NES-CHIP spent less time climbing down the pole and had longer latencies in falling from the accelerating rod (Figure 7A, 7B, 7D, 7E). In the open-field test, mice with overexpressed CHIP crossed more squares within 5 min, reflecting better spontaneous activity (Figure 7C, 7F). The comparisons that reached statistical significance are indicated in the figures.

### CHIP overexpression rescued MPTP-induced dopaminergic degeneration in vivo

**Figure 8:**
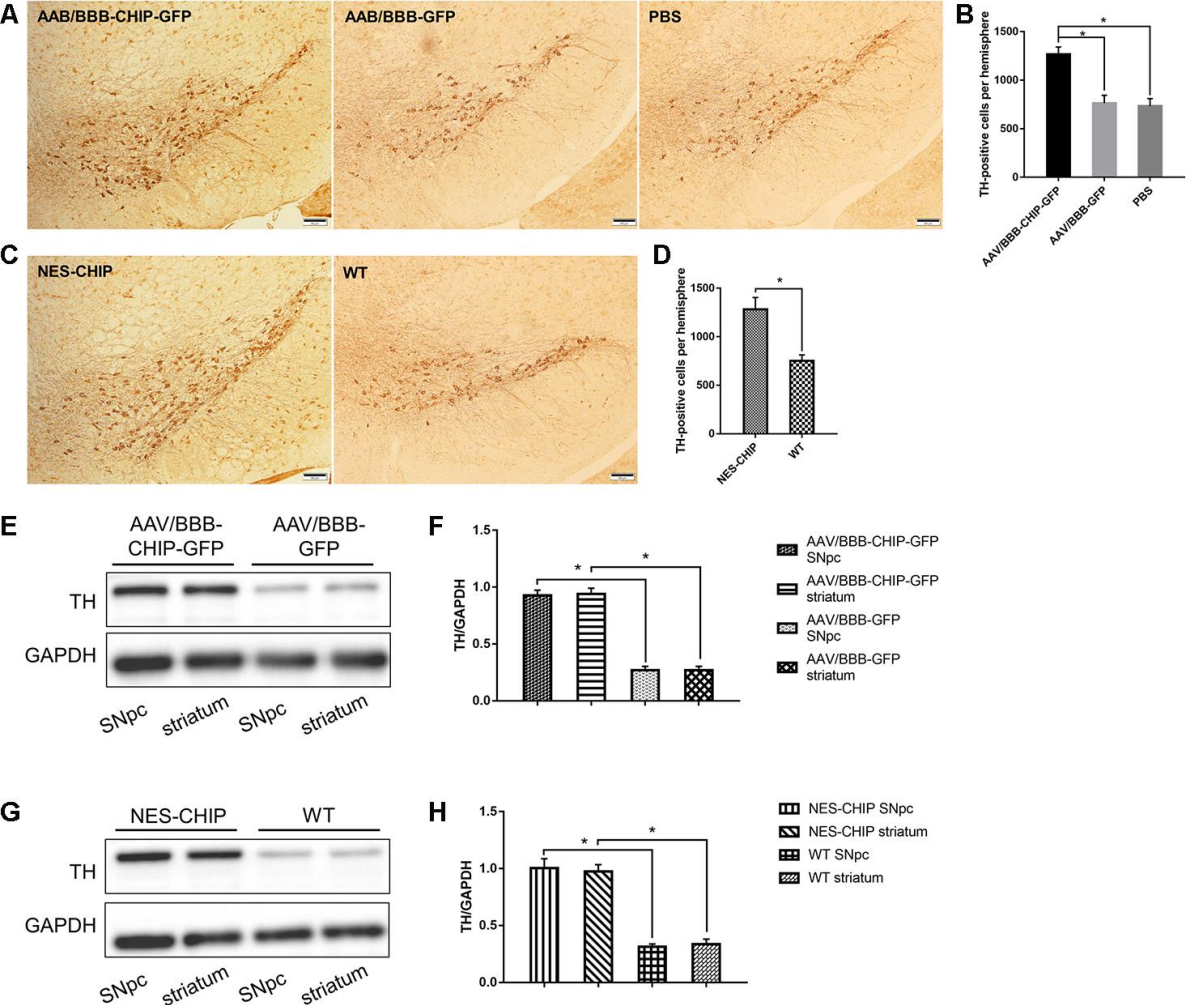
**Immunohistochemistry of TH-positive cells and determine of TH protein.** (**A**–**D**) Representative images of dopaminergic neurons stained for tyrosine hydroxylase (TH) in midbrain sections of different groups of mice. MPTP caused a significant decrease of TH-positive cells, while CHIP overexpression rescued the cell loss. (**E**–**H**) TH protein levels in SNpc and striatum of overexpression groups were significantly higher than those in control groups. *P<0.05.

Progressive loss of dopaminergic neurons is one of the main pathological features of PD. Tyrosine hydroxylase (TH) is the marker of dopaminergic neurons, and the abundance of TH-positive cells in the SNpc and striatum can reflect the severity of neuronal loss after MPTP exposure. Immunohistochemistry results showed that CHIP protected TH-positive cells from MPTP damage. Cell counting confirmed a statistical difference (Figure 8A–8D). According to the immunoblotting results, TH protein levels decreased significantly in mice pre-treated with AAV/BBB-GFP or vehicle relative to those with AAV-mediated CHIP overexpression (Figure 8E, 8F). Similar results were confirmed in NES-CHIP mice and wild-type littermates (Figure 8G, 8H).

### CHIP overexpression prevented an acute increase in Drp1 in the MPTP-induced PD mouse model

**Figure 9:**
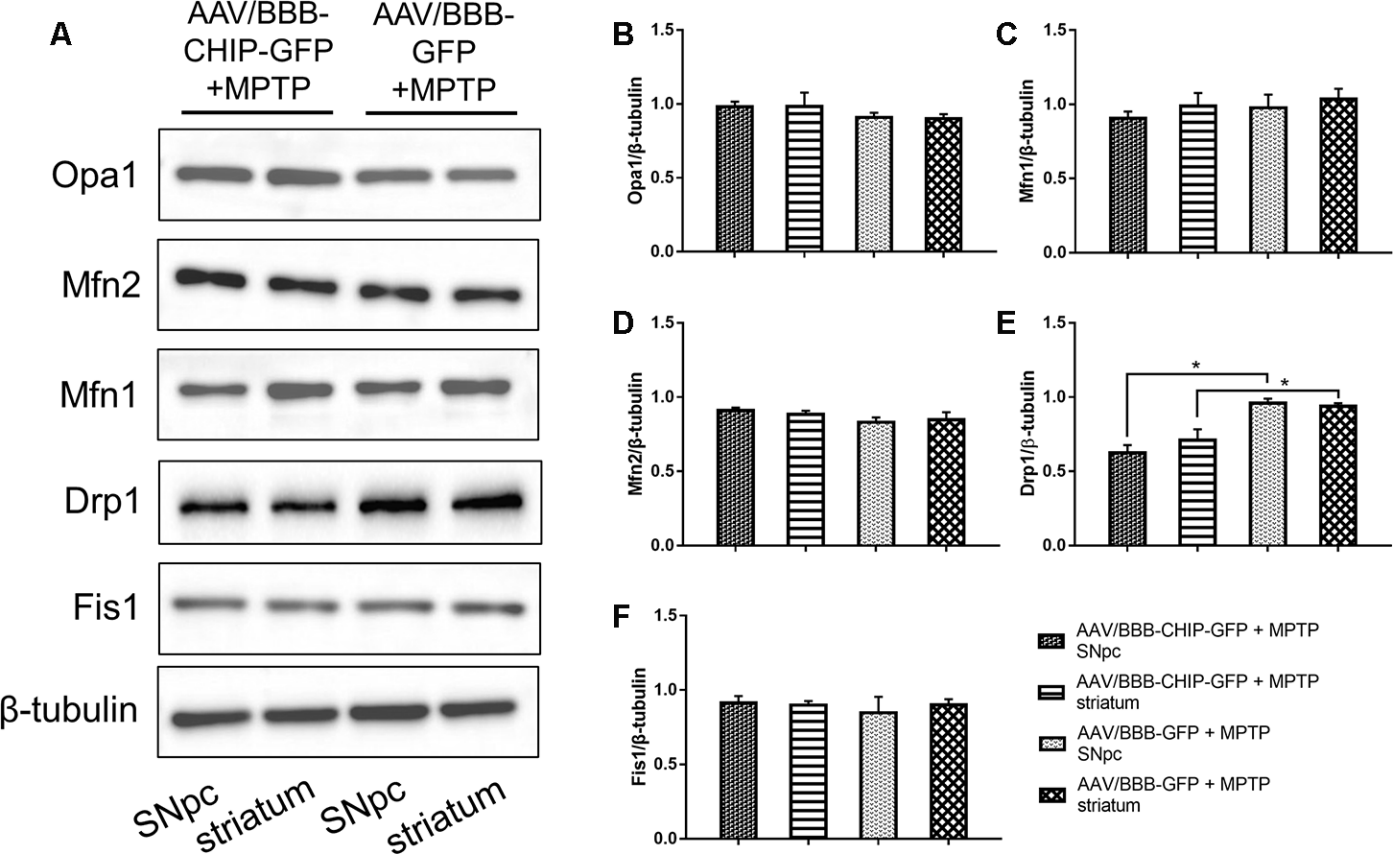
**Western blotting of proteins about mitochondrial fusion and fission in SNpc and striatum in mice treated with AAV/BBB-CHIP-GFP or AAV/BBB-GFP followed by MPTP administration.** Statistical analysis showed that CHIP increased in AAV/BBB-CHIP-GFP injected mice. (**A**) Drp1 was upregulated after MPTP administration. CHIP overexpression inhibited the rising trend of Drp1. (**B**–**F**) Quantitative analysis of the blots. *P<0.05.

**Figure 10:**
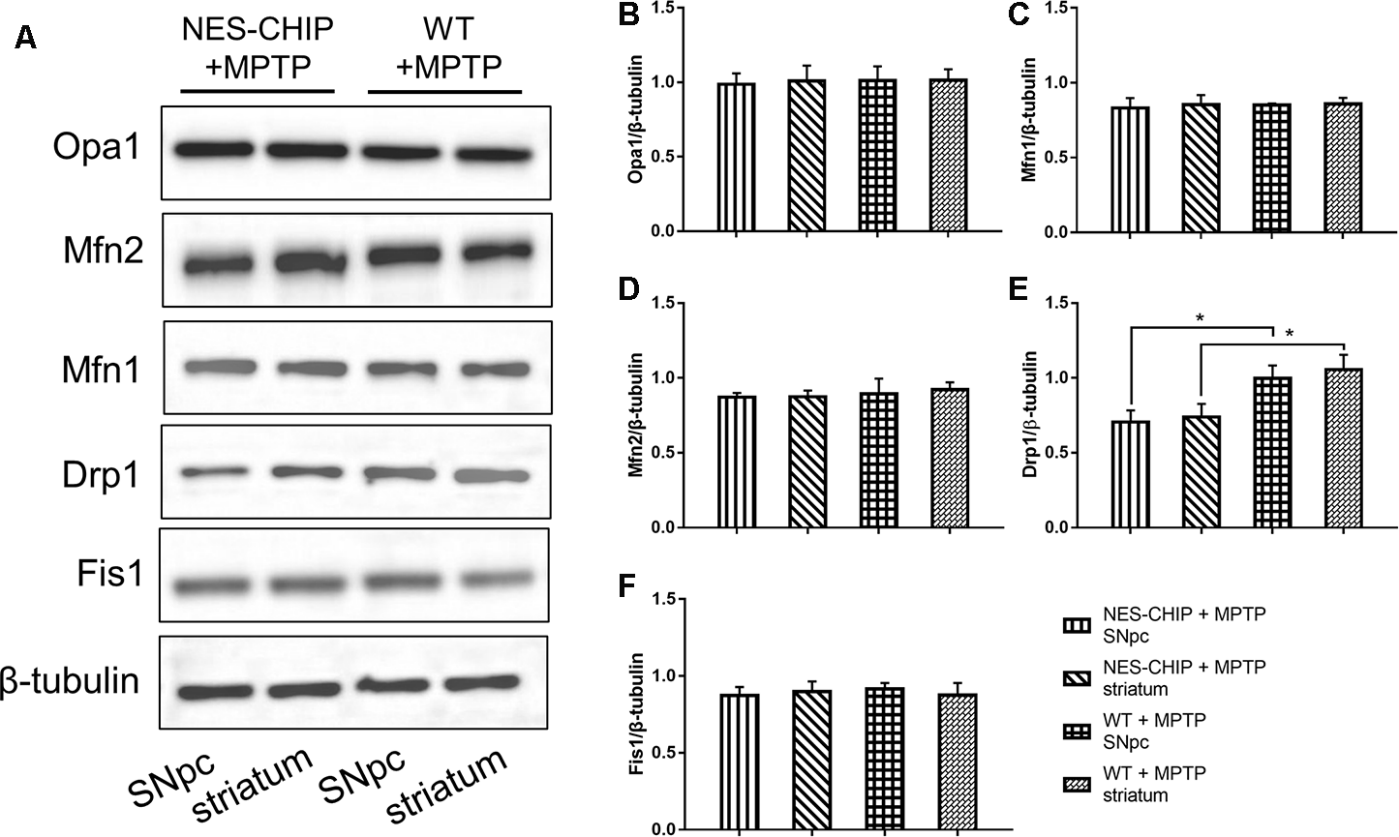
**In MPTP-treated NES-CHIP mice and the littermates wild-type mice, proteins in SNpc and striatum were detected by Western blot.** (**A**) Under the action of MPTP, the level of Drp1 increased significantly in wildtype mice than that in NES-CHIP littermates. (**B**–**F**) Quantitative analysis of the blots. *P<0.05.

The typical morphology of mitochondria reflects a dynamic equilibrium between fusion and fission. We investigated if CHIP affects the level of proteins related to these two processes. Mice were sacrificed on the 7^th^ day after the last injection of MPTP. The SNpc and striatum were isolated and total proteins were extracted. The five targets assessed by western blot included the following fusion- and fission-associated proteins: Opa1, Mfn1, Mfn2, Drp1, and Fis1. Seven days after the end of MPTP administration, Drp1 levels in the AAV/BBB-GFP or vehicle pre-treated mice were significantly higher than those in the AAV/BBB-CHIP-GFP pre-treated mice (Figure 9). In the NES-CHIP mice, Drp1 levels post-MPTP were lower than those in their wild-type littermates (Figure 10). No difference in protein levels was observed between the SNpc and striatum. These results indicated that CHIP regulates mitochondrial morphology through Drp1, thus protecting neurons against MPTP-induced toxicity.

## DISCUSSION

This study explored the protective role of CHIP in cell and animal PD models. We could examine cells having both raised and lowered CHIP levels using different plasmids to transfect the SH-SY5Y cells. When the endogenous CHIP was knocked out, the cell damage and apoptosis induced by MPP^+^ were exacerbated, while CHIP overexpression prevented cell damage. Furthermore, our results showed that CHIP overexpression attenuated the motor deficits and the loss of dopaminergic neurons from the SNpc and striatum caused by MPTP. No differences in motor function were observed between mice with CHIP overexpression or not. *In vitro and in vivo* experiments demonstrated that these protective effects were achieved by CHIP-mediated decreases in the levels of Drp1. Taken together, our results indicate that upregulation of CHIP is protective against MPP^+^/MPTP-induced neurotoxicity.

Mitochondria are dynamic organelles that form networks in cells. The abundance, morphology, and cellular localization of mitochondria can all influence mitochondrial homeostasis [17]. Disruption of mitochondrial quality control contributes to various neurodegenerative diseases [18, 19] and reductions in the activity of the mitochondrial respiratory chain complex I caused by MPP^+^/MPTP have been associated with PD [20]. Moreover, some of the gene variants implicated in PD pathogenesis have been linked to mitochondrial damage [21–23]. *LRRK2* mutations are the main cause of familial PD, and studies in *C. elegans* and cultured neurons have shown that *LRRK2* mutations alter mitochondrial dynamics. Furthermore, mutations in the PD-associated *SNCA* gene can cause increased mitophagy and mtDNA damage [24, 25].

The processes of fusion and fission determine mitochondrial morphology, and the balance between the two opposing processes is fundamental to the maintenance of normal mitochondrial function [26]. The central nervous system (CNS) consumes a considerable amount of energy, and neurons require functional mitochondrial networks to supply energetically demanding cellular activities such as synaptic transmission and axoplasmic transport [27, 28]. The primary regulators of mitochondrial fission are Drp1 and Fis1, whereas fusion is controlled mainly by Mfn1, Mfn2, and Opa1 [29]. Of the fission- and fusion-associated proteins, Drp1 plays a major role in regulating the normal shape of mitochondria as well as in regulating growth and development [17, 30]. Drp1 belongs to the guanosine triphosphatase (GTPase) family and regulates several intracellular molecular pathways [31]. Mice with Drp1 deficiencies exhibit neuronal defects and impaired synapse formation, and mutations in Drp1 have been linked to abnormal brain development [32–34].

Drp1 is involved in the pathogenesis of PD. In an *in vitro* model of PD established by overexpressing α-synuclein, mitochondrial fragmentation induced by the effects of α-synuclein depended on Drp1 [35]. Drp1 also mediated the apoptosis and necroptosis process in PD. Inhibition of Drp1 is considered a novel treatment method in PD. In *PINK1* knock-out mice, the negative mutant Drp1-K38A decreased the expression of Drp1 and restored dopamine release [36]. Application of MPP^+^/MPTP caused abnormal mitochondrial homeostasis and increased Drp1, and a Drp1 inhibitor evidently plays a neuroprotective role in PD models [37, 38]. Our data demonstrated increased Drp1 after MPP^+^ treatment in the CHIP knockdown condition, and the mitochondrial division inhibitor 1 (Mdivi-1) prevented this rising trend in Drp1 level. Moreover, the results of MitoTracker Red staining showed less mitochondrion fragmentation after MPP^+^ treatment in the CHIP overexpression group.

Recombinant adeno-associated viruses are useful vectors in research, as they do not integrate into the host genome. AAV/BBB is a new serotype of AAV that can cross the blood-brain barrier with enhanced efficiency, and is based on the work by Gradinaru et al. on AAV-PHP.B, which is a capsid variant of AAV9 that can transfer intravenously administered genes throughout the CNS with greater efficiency than the current serotype [39]. Though a previous study showed that enhanced CNS tropism is limited to the transgenic mouse model chosen, diverse mouse strains reportedly share similar neurotropic properties when they are injected with AAV-PHP.B. In research on Pompe disease, a neuromuscular disorder caused by a deficiency of acid α-glucosidase (GAA), AAV-PHP.B-mediated gene therapy demonstrated efficacy in treating GAA-knockout mice. In addition, the AAV-treated knockout mice reportedly had decreased levels of glycogen in the brain, heart, and skeletal muscles [40]. However, similar results in neurodegeneration models have not been previously reported. Our experiments confirm once again that the enhanced ability of AAV to cross the blood-brain barrier renders it useful in the study of neurological diseases.

In this study, we demonstrated that overexpression of CHIP could prevent MPP^+^-induced damage in SH-SY5Y cells and ameliorate the motor function of MPTP-treated mice by regulating Drp1, a molecule functionally downstream from CHIP. Further studies are required to clarify the interaction between CHIP and Drp1 as well as the role of Drp1 in the pathogenesis of PD. In addition, our results support further investigation of gene therapy for neurodegenerative diseases, including PD.

## MATERIALS AND METHODS

### Cell culture

### Generation of the MPP^+^-induced cell model

The human neuroblastoma cell line SH-SY5Y was purchased from the American Type Culture Collection (ATCC, Manassas, VA, USA). Cells were used and maintained in Dulbecco’s modified Eagle’s medium (Hyclone, South Logan, UT, USA) supplemented with 10% fetal bovine serum (Biological Industries, Kibbutz Beit Haemek, Israel) and cultured at 37° C in a moist atmosphere containing 5% CO_2_. To obtain the *in vitro* experimental PD model, 0.5, 1, 2, and 3 mM of MPP^+^ (Sigma-Aldrich, St. Louis, MO, USA) were added to different wells, which were then cultured for 24 h to determine the optimal concentration of MPP^+^.

### Plasmid and transfection

SH-SY5Y cells were transfected with the CHIP-WT plasmid (1 μg per 1 × 10^5^ cells) using Lipofectamine^TM^ 2000 (Invitrogen, Carlsbad, CA, USA) to induce overexpression of human CHIP. To generate the CHIP knockdown and control groups, cells were transfected with an shRNA (5’-GGCCTTGTGCTACCTGAAGAT-3’) against human CHIP and a scrambled control shRNA with no significant homology to any known gene sequence, respectively. The plasmid and shRNA were made by GenePharma (Shanghai, China). After 6 h of incubation, the medium was replaced with regular culture medium, MPP^+^ was added, and the cells were cultured for an additional 24 h.

### Treatment with Mdivi-1

We added 10 mM of mitochondrial fission inhibitor 1 (Mdivi-1, MedChem Express, Monmouth Junction, NJ, USA) dissolved in DMSO to an SH-SY5Y cell suspension 6 h after transfection, when the MPP^+^ was added. After a further 24 h of incubation, cell viability, LDH levels and ATP generation were measured.

### Cell viability assay

After the completion of the indicated experiments, Cell Counting Kit 8 (CCK8, DojinDo, Kumamoto, Japan) was added to each well and incubated for 1 h at 37° C. The optical density at 450 nm was then measured using a microplate reader to index cell viability. Each experiment was repeated thrice.

### LDH cell damage assay

An LDH cytotoxicity assay kit (Beyotime, Shanghai, China) was used to assay the amount of LDH released from damaged cells. Absorbance at 490 nm was used to quantify the amount of LDH. The ratio of LDH activity in the supernatant to total LDH activity was used to index cell death as outlined in the manufacturer’s instructions.

### Measurement of ATP

The level of ATP in SH-SY5Y cell lines under different conditions was determined with an ATP bioluminescence assay kit (Beyotime, Shanghai, China). ATP concentration was determined by phosphorylating glycerol, resulting in a colorimetric product with an absorption at 570 nm, which is proportional to the amount of ATP present.

### Mitochondrial morphology

SH-SY5Y cells were loaded with preheated, 100 nM MitoTracker Red CMXRos (Meilun Bio, Dalian, China) prepared with complete medium. After incubation at 37° C for 30 min, the culture medium was replaced with fresh medium and the cells were live-imaged with the Leica Multiphoton system.

### Animal experiments

This study was approved by the Life Science Ethics Review Committee of Zhengzhou University.

CHIP-with-floxed-STOP-codon mice were generated with CRISPR/Cas9 (Biocytogen Co., Ltd (Beijing, China). The CHIP knock-in and wild-type mice both had a C57BL/6 genetic background. Mice with the CHIP-with-floxed-STOP codon were crossed with Nestin-cre mice (Jackson Labs Technologies, West Grove, PA, USA) to yield NES-CHIP animals. All the animals were housed in a specific-pathogen–free environment with a 12/12-h light/dark cycle and free access to water and food. The temperature and humidity were controlled at 22 ± 1° C and 60 ± 5%, respectively. Only male mice were used in the experiments.

### Pole test

Movement disorders were assessed using the pole test. The apparatus was a wooden pole with a height of 60 cm and a width of 1 cm, with a 2-cm diameter wooden ball affixed to the top. The pole was covered in gauze to prevent sliding. Each mouse was placed on the wooden ball, and the total time taken for the mouse to climb down the pole was measured. Pre-training was necessary to ensure that the mice attempted to move down the pole after they were placed on the top.

### Rotarod test

Motor coordination and learning ability were evaluated using an accelerating rotarod test. The mouse was placed on an automatic rotarod bar with a diameter of 3 cm, and the apparatus then accelerated from 4 to 40 rpm within 5 min. Each test comprised three trials with an interval of 1 h between trials. The latency to falling from the rod was recorded. Before drug treatment, mice were trained for three consecutive days to ensure that they could remain on the rod for 5 min at baseline.

### Open-field test

Spontaneous locomotor activity was analyzed using an open-field test. The apparatus consisted of a square arena and surrounding walls, and measured 40 × 40 × 40 cm. The floor was divided into 16 squares. Each mouse was placed in the central square of the arena, and the number of squares that the mouse passed over within 5 min was recorded. The arena was wiped with alcohol after each test.

### Drugs and treatments

AAV/BBB-CHIP-GFP and the control AAV/BBB-GFP were made by Hanbio Biotechnology (Shanghai, China). When the male wild-type mice reached 6–8 weeks of age, 100 μL of AAV were injected into the tail vein. At 10–12 weeks of age, the AAV-injected mice, the NES-CHIP mice, and the wild-type mice received four intraperitoneal injections of 20 mg/kg of MPTP (Sigma-Aldrich, St. Louis, MO, USA) or 0.9% phosphate-buffered saline (PBS) at 2-h intervals. Our experiment thus featured five groups of animals: AAV/BBB-CHIP-GFP, AAV/BBB-GFP, vehicle (PBS), NES-CHIP, and wild-type. 8 to 10 mice were included in each group. Behavioral testing was conducted at baseline and on the 1^st^, 3^rd^, 5^th^, and 7^th^ days after MPTP injection.

### Tissue preparation

Mice were sacrificed under anesthesia at day 7 following the last injection. For immunohistochemistry, the mice were perfused successively with 1 × PBS and 4% paraformaldehyde (Servicebio, Wuhan, Hubei, China), and the whole brain was removed by craniotomy. The mice brains were post-fixed in 4% PFA overnight and dehydrated with 20% and 30% sucrose solution at 4° C. Coronal slices were cut at a thickness of 20 μm with a freezing microtome (Leica, Wetzlar, Germany) and stored at -20° C. For immunoblotting, the brains were separated into different regions and frozen at -80° C.

### Immunohistochemistry and immunofluorescence

The frozen brain slices were returned to room temperature and washed thrice in PBS to remove the tissue freezing medium, and antigens were retrieved. A SPlink Detection Kit was used for immunohistochemistry (Zsbio, Beijing, China). Endogenous peroxidase was blocked by incubation with 0.3% H_2_O_2_, and nonspecific sites were blocked with 5% normal sheep serum in 0.3% triton X-100. The slices were incubated overnight at 4° C with the primary antibody, anti-tyrosine hydroxylase (#AB152, Millipore, Billerica, MA, USA). On the following day, the slices were incubated with secondary antibody at room temperature. Diaminobenzidine (DAB) was used to visualize antibody binding. For immunofluorescence, the slices were incubated with the primary anti-CHIP (55430-1-AP, Proteintech, Wuhan, Hubei, China) and anti-GFP antibodies (66002-1-Ig, Proteintech) under the aforementioned conditions. The secondary antibodies were tagged with different fluorescent labels. Cell nuclei were stained with DAPI (Solarbio, Beijing, China).

### Western blot

Total protein was extracted from brain tissue or cultured cells with ice-cold RIPA buffer and protease inhibitor mixture (Solarbio). Equal amounts of protein per track were loaded onto 10% SDS-PAGE gels (Epizyme, Shanghai, China) for electrophoresis and then transferred onto PVDF membranes (Millipore) at low temperature. After blocking in 5% non-fat milk, the membranes were incubated overnight with primary antibody followed by incubation with horseradish peroxidase (HRP)-conjugated secondary antibody. The primary antibodies included anti-GAPDH (#10494-1-AP, Proteintech), anti-beta tubulin (#10094-1-AP, Proteintech), anti-TH (#AB152, Millipore), anti-CHIP (#2080, CST; Danvers, MA, USA), anti-Opa1 (27733-1-AP, Proteintech), anti-Drp1 (12957-1-AP, Proteintech), anti-Mfn1 (#13798-1-AP, Proteintech), anti-Mfn2 (#12186-1-AP, Proteintech), and anti-Fis1 (#10956-1-AP, Proteintech). Membranes were developed using the ECL luminescence reagent (#SQ101, Epizyme) and the protein bands visualized by enhanced chemiluminescence. Signal intensities were quantified with Image J software (NIH, Bethesda, MD, USA) and normalized to an internal control protein.

### Statistical analysis

The data are expressed as mean ± standard deviation (SD) and analyzed with SPSS21.0 statistical software (IBM, Armonk, NY, USA). Student’s *t*-tests were used to compare two groups, and one-way analysis of variance (ANOVA) was used for comparisons of multiple groups. The level of statistical significance was set to *P* < 0.05.
